# Sex- and strain-differential plasma proteomic signatures in C57BL/6 and BALB/c mice

**DOI:** 10.1038/s41598-025-23706-4

**Published:** 2025-11-14

**Authors:** Yunha Suh, Kwang-eun Kim

**Affiliations:** 1 Organelle Medicine Research Center, Yonsei University Wonju College of Medicine, Wonju, 26426 Republic of Korea; 2 Department of Convergence Medicine, Yonsei University Wonju College of Medicine, Wonju, 26426 Republic of Korea; 3 Department of Global Medical Science, Yonsei University Wonju College of Medicine, Wonju, 26426 Republic of Korea

**Keywords:** Biochemistry, Biological techniques, Biomarkers, Computational biology and bioinformatics, Diseases, Molecular biology

## Abstract

Sex is a critical determinant of health and disease, yet it remains underrepresented in biomedical research. The identification of blood-based biomarkers facilitates early diagnosis and intervention for various diseases; however, sex-differential differences in the plasma proteome have not been sufficiently explored in mouse models. Understanding the molecular features associated with sex is essential for enhancing the translational potential of clinical research. We utilized Olink technology to analyze sex- and strain-differential plasma protein expression in two widely used mouse strains, C57BL/6 and BALB/c. A total of 36 mice (n = 9 per strain and sex) were analyzed using the ‘Olink Target 48 Mouse Cytokine’ and ‘Olink Target 96 Mouse Exploratory’ panels. Differences in normalized protein expression (NPX) were compared between groups, and proteins with a P-value < 0.05 were considered significantly different. Our analysis identified 55 strain-differential proteins and 33 sex-differential proteins among the 87 proteins analyzed in mouse plasma. Importantly, LPL (Lipoprotein lipase) and GHRL (Appetite-regulating hormone) were also more highly expressed in females in human datasets, suggesting a conserved sex-biased expression pattern across species. This study characterized sex- and strain-differential differences in the plasma proteomes of C57BL/6 and BALB/c mice. Among the identified proteins, LPL and GHRL were significantly elevated in females, consistent with human gene and plasma protein expression trends. These findings highlight the presence of sex-based molecular differences in energy and lipid metabolism and provide a valuable foundation for future mechanistic studies.

## Introduction

Proteins play essential roles in living organisms and directly reflect physiological and pathophysiological processes in the human body, making them widely used as biomarkers^1^. Among them, blood-based biomarkers are particularly valuable. Since plasma proteins originate from a variety of surrounding tissues^2^, the plasma proteome serves as an integrative indicator of the body’s overall physiological state. Moreover, blood-based biomarkers can serve as early indicators of disease risk and onset during asymptomatic stages^3^, allowing for timely intervention. However, reliable blood-based biomarkers that can accurately predict disease development remain insufficient.

One critical factor that has been overlooked in biomarker research is sex differences. Sex is a fundamental biological variable that encompasses genetic, hormonal, and physiological differences. Previous studies have reported sex-based disparities in disease incidence, progression, and treatment responses in humans^4–7^. For example, autoimmune diseases are well-known for their sex-biased prevalence^8^, and Alzheimer’s disease (AD) also exhibits marked sex differences^9^. In addition, an increasing number of studies are investigating sex-differential patterns in various cancers^4,5,10–13^. These findings collectively support the view that sex can influence disease mechanisms and therapeutic responses.

Between 2000 and 2021, the United States, Canada, and the United Kingdom, implemented funding policies to promote sex and gender research, leading to notable improvements in the quality and publication rates of such studies^14^. Despite this, sex-based differences have often been underrecognized in biomedical research. A study from 2011 reported that male animals were used 5.5 times more often than females in preclinical research^15^. This imbalance is partly due to concerns that hormonal fluctuations during the female reproductive cycle could introduce variability into experimental outcomes^16^. However, subsequent evidence has shown that behavioral variability in female rodents is not significantly greater than in males^17,18^. Still, research remains predominantly skewed toward one sex, limiting the generalizability and inclusiveness of scientific findings.

In the drug development process, most candidate compounds are tested for efficacy and side effects using animal models^19^, with various mouse models playing a central role^20^. However, even if a drug shows success in mouse experiments, unexpected adverse effects may arise during clinical trials. One potential reason for this discrepancy is that preclinical studies predominantly use male mice, often overlooking potential sex-based biological differences^21^. A significant number of studies either fail to report the sex of animal or cell models or omit sex-based analyses altogether, undermining their translational potential in clinical settings. Addressing this gap is essential to improve the predictive value of mouse studies and to ensure the development of more effective and inclusive therapeutic strategies.

In addition to sex differences, genetic variations are also important. A study comparing multiple mouse strains reported differences related to immune responses, sensory functions, and behavioral traits^22^. For example, when allergy was induced by exposing the airway to ovalbumin, BALB/c mice exhibited a stronger antibody response compared to C57BL/6 mice^23^. In the human study, differences in the prognosis of metabolic diseases were observed among different racial groups^24^. Another study reported that racial and ethnic disparities in tuberculosis incidence persisted even after adjusting for age and sex^25^. Another example is the drug BiDil, which was the first heart failure medication approved specifically for Black patients^26^. These findings demonstrate that physiological responses to the same stimulus or drug can vary significantly depending on the genetic difference or mouse strain.

In conclusion, incorporating sex and strain as variables in the analysis is essential for enhancing the accuracy of result interpretation and preventing misleading conclusions^27^. Therefore, it is critical to consider both biological diversities—sex and strain—in studies aiming to identify plasma biomarkers. In this context, the present study aimed to investigate the effects of sex and strain differences on plasma protein expression in C57BL/6 and BALB/c mice using high sensitivity Olink proteomics.

## Methods

### Animals and blood collection

All animal procedures were approved by the Institutional Animal Care and Use Committee (IACUC) of Yonsei University Wonju College of Medicine. The authors confirm that this study is reported in accordance with the ARRIVE guidelines and that all relevant recommendations were followed. Mice were obtained from Orient Bio Inc. (Seongnam, Republic of Korea). Four groups of 8-week-old mice were used in the study: C57BL/6N males (n = 9), C57BL/6N females (n = 9), BALB/c males (n = 9), and BALB/c females (n = 9). The mice were in the fed state at the time of blood collection. The mice were euthanized by exposure to CO₂, and no additional anesthesia was administered. Blood was collected via cardiac puncture and transferred into EDTA-coated microtubes (BD, Cat# 365,974). After gentle inversion, samples were centrifuged at 5,000 × g for 15 min at 4 °C. The supernatant plasma was carefully collected and stored at − 80 °C until analysis.

### Olink analysis

Plasma protein levels were measured using the ‘Olink Target 48 Mouse Cytokine (#93,400)’ and the ‘Olink Target 96 Mouse Exploratory (#95,380)’ panels. Two panels were analyzed by DNA Link Inc. (Seoul, Republic of Korea) or by Macrogen Inc. (Seoul, Republic of Korea). Protein expression levels were quantified using Olink’s NPX (Normalized Protein Expression) values, which are presented on a log_2_ scale. For more intuitive interpretation, NPX values were converted to 2^NPX^ for data analysis.

### Human protein atlas dataset

Publicly available Olink data from a two-year longitudinal health study were utilized^28^. During the study period, 76 participants (40 males and 36 females) visited the clinic at three time points: visit 1 (0 months), visit 2 (15–18 months), and visit 3 (21–24 months). All participants were fasting overnight or at least 8 h before the visits. The expression levels of 1,463 proteins were measured and normalized using the NPX scale. Protein expression levels were presented separately for male and female groups^28^. These results are integrated into the Blood section of the Human Protein Atlas under Longitudinal analysis of blood protein levels (two years). RNA expression levels were extracted from the human RNA-Seq data of GTEx dataset. No separate record was available regarding fasting status. RNA-seq data across 36 tissue types were mapped using RSEM v1.3.0 (v8), and transcript abundance was expressed in nTPM (Normalized Transcripts Per Million). The resulting nTPM values were incorporated into the Tissue section of the Human Protein Atlas.

### Statistics

Statistical analysis was performed using the two-way ANOVA. P-values of less than 0.05 were considered statistically significant.

## Results

### Overview of experimental design

**Fig. 1:**
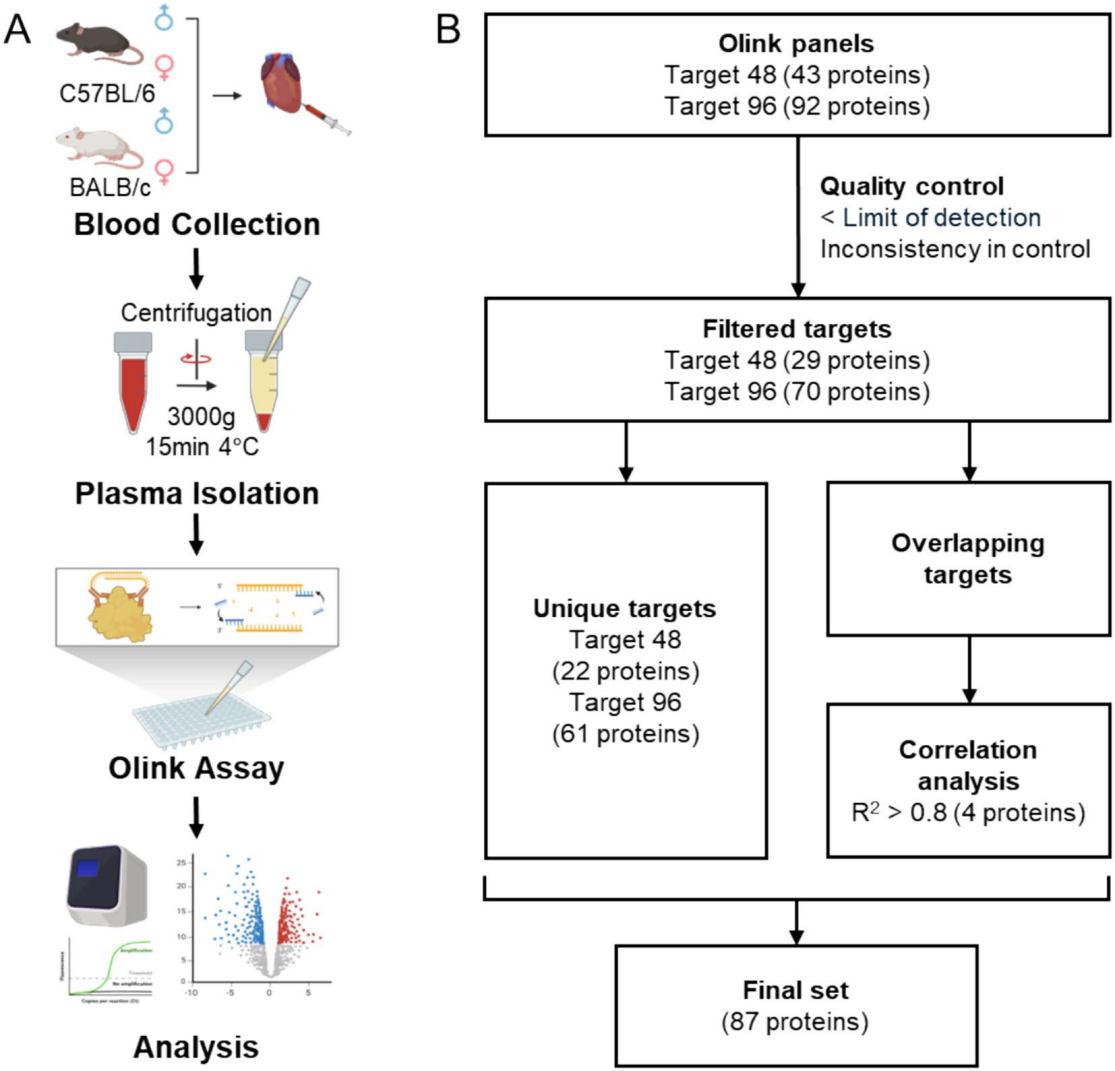
Experimental scheme and data analysis workflow for investigating sex differences in mouse plasma. **(A)** Experimental scheme. Plasma was collected from mice and subjected to Olink assay, followed by data analysis. **(B)** Data analysis workflow. The criteria used to select the final set of 87 proteins are depicted. Created with BioRender.com.

We collected blood samples via cardiac puncture from male and female mice of two strains and subsequently isolated plasma for Olink analysis **(**Fig. 1A**)**. Two Olink panels (Target 48 Mouse Cytokine Panel and the Target 96 Mouse Exploratory Panel) were used in this study **(**Fig. 1B**)**. Proteins with values below the limit of detection or those showing inconsistencies in control samples (including negative controls and calibrators for quality assurance) were excluded from the analysis. For overlapping targets between the two panels, correlation analyses were conducted, and four proteins showing consistent correlations were selected. In total, sex- and strain-differential differences were examined across a final set of 87 target proteins.

### Identification of strain-differential plasma proteins

**Fig. 2:**
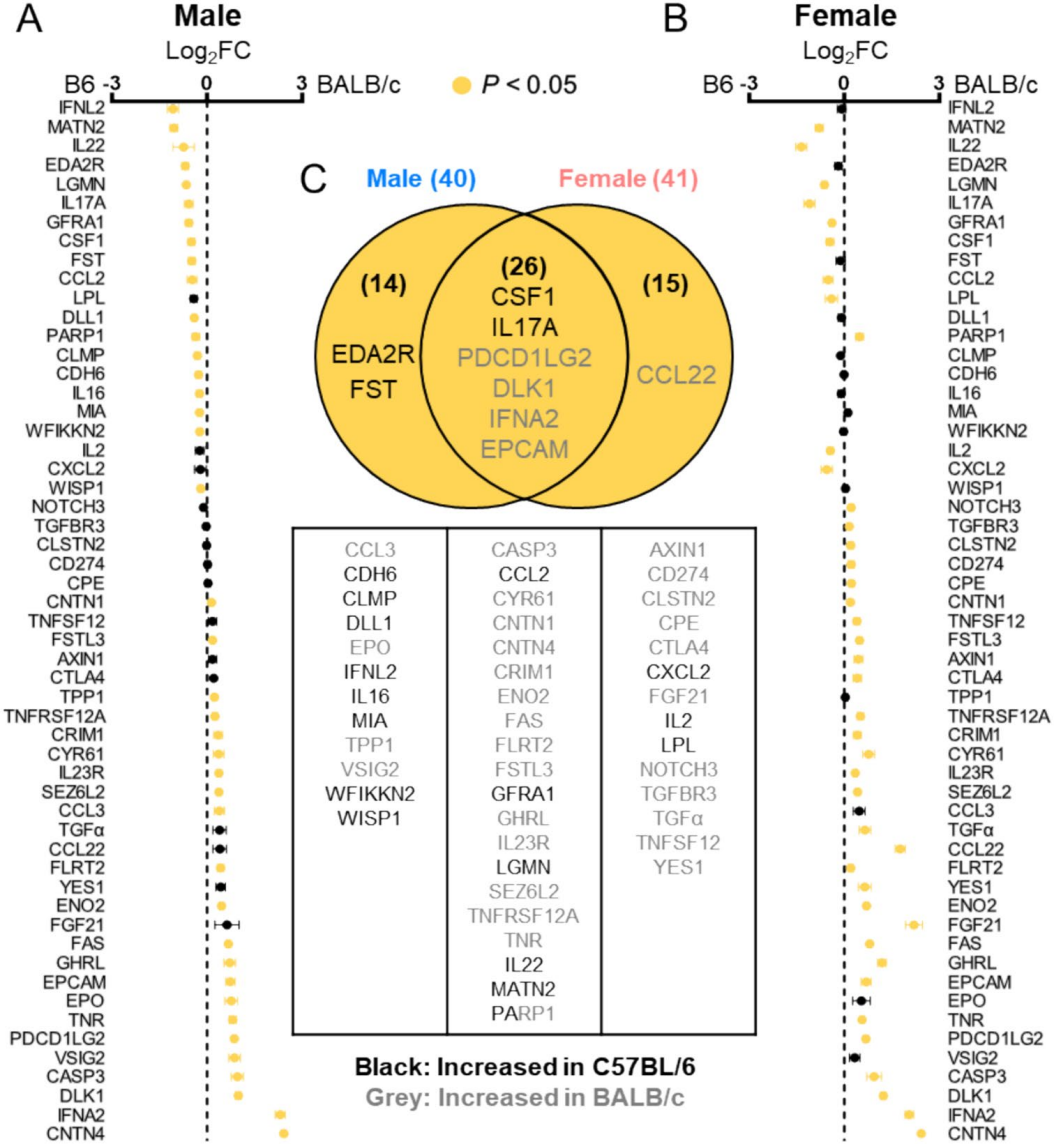
Strain-differential proteins in plasma. **(A, B)** Fold changes (FC) were calculated based on NPX values obtained from the Olink assay. **(A)** Comparison between C57BL/6 (B6) male and BALB/c male. **(B)** Comparison between C57BL/6 male and BALB/c female. Proteins with statistically significant differences (*P* < *0.05*) are highlighted in yellow. **(C)** Venn diagram and list illustrating the number and overlap of proteins showing strain differences in male and female mice.

**Fig. 3:**
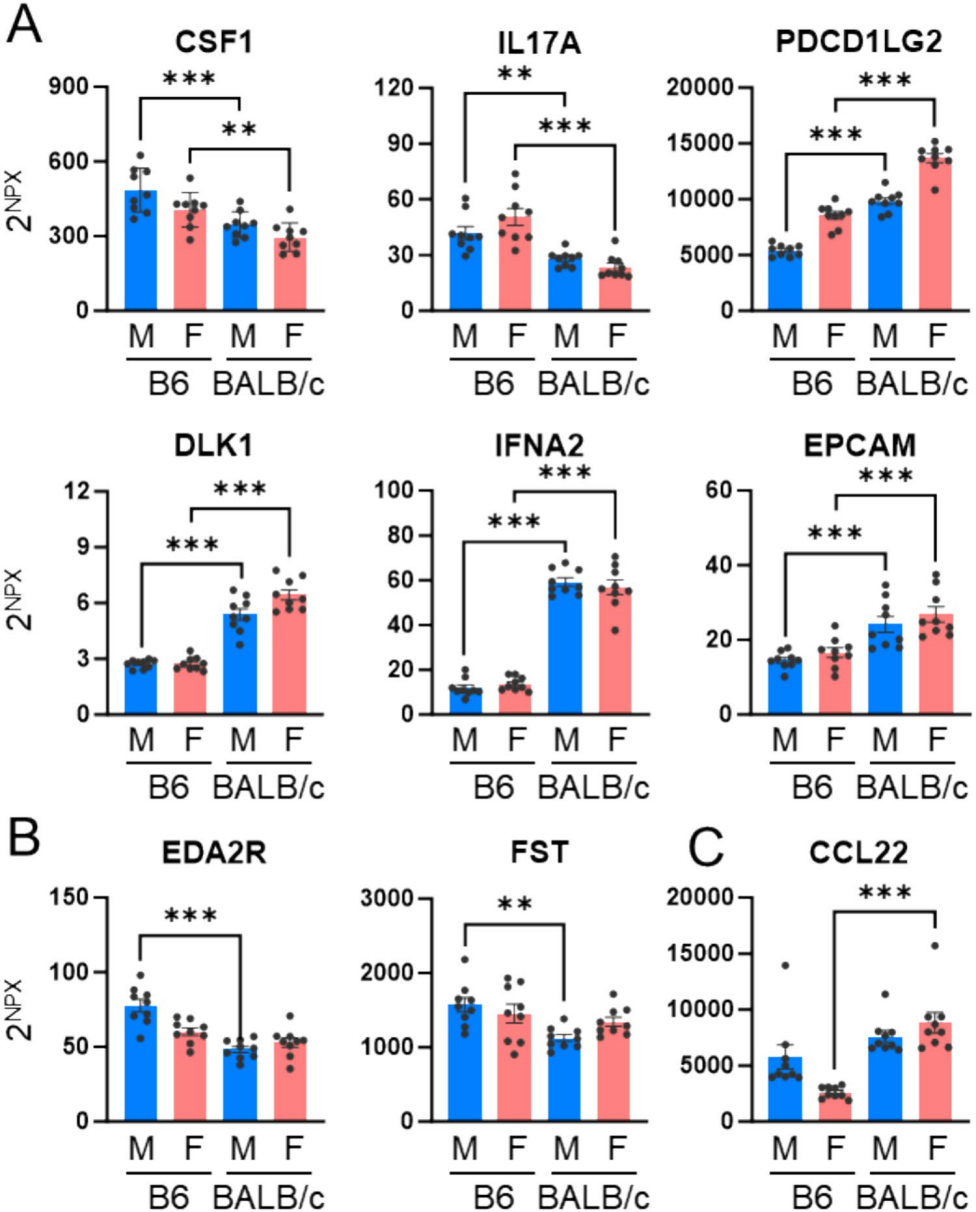
Representative plasma proteins that exhibit strain-differential expression differences. **(A)** Proteins showing strain differences in both males (M) and females (F). **(B)** Proteins showing strain differences only in males. **(C)** Proteins showing strain differences only in females. The data are denoted as the mean ± SEM. **P < 0.01; ***P < *0.001.*

Among the 87 analyzed proteins, 55 (63.2%) exhibited strain-differential expression. 40 proteins showed differential expression in males **(**Fig. 2A**)**, and 41 in females **(**Fig. 2B**)**. A total of 26 proteins were commonly affected by strain in both sexes **(**Fig. 2C**)**. Proteins consistently elevated in the same strain across both sexes included CSF1, IL17A, PDCD1LG2, DLK1, IFNA2, and EPCAM **(**Fig. 3A**)**. Proteins showing strain-differential expression only in males included EDA2R and FST **(**Fig. 3B**)**, while CCL22 showed differential expression in females **(**Fig. 3C**)**.

### Identification of sex-differential plasma proteins

**Fig. 4:**
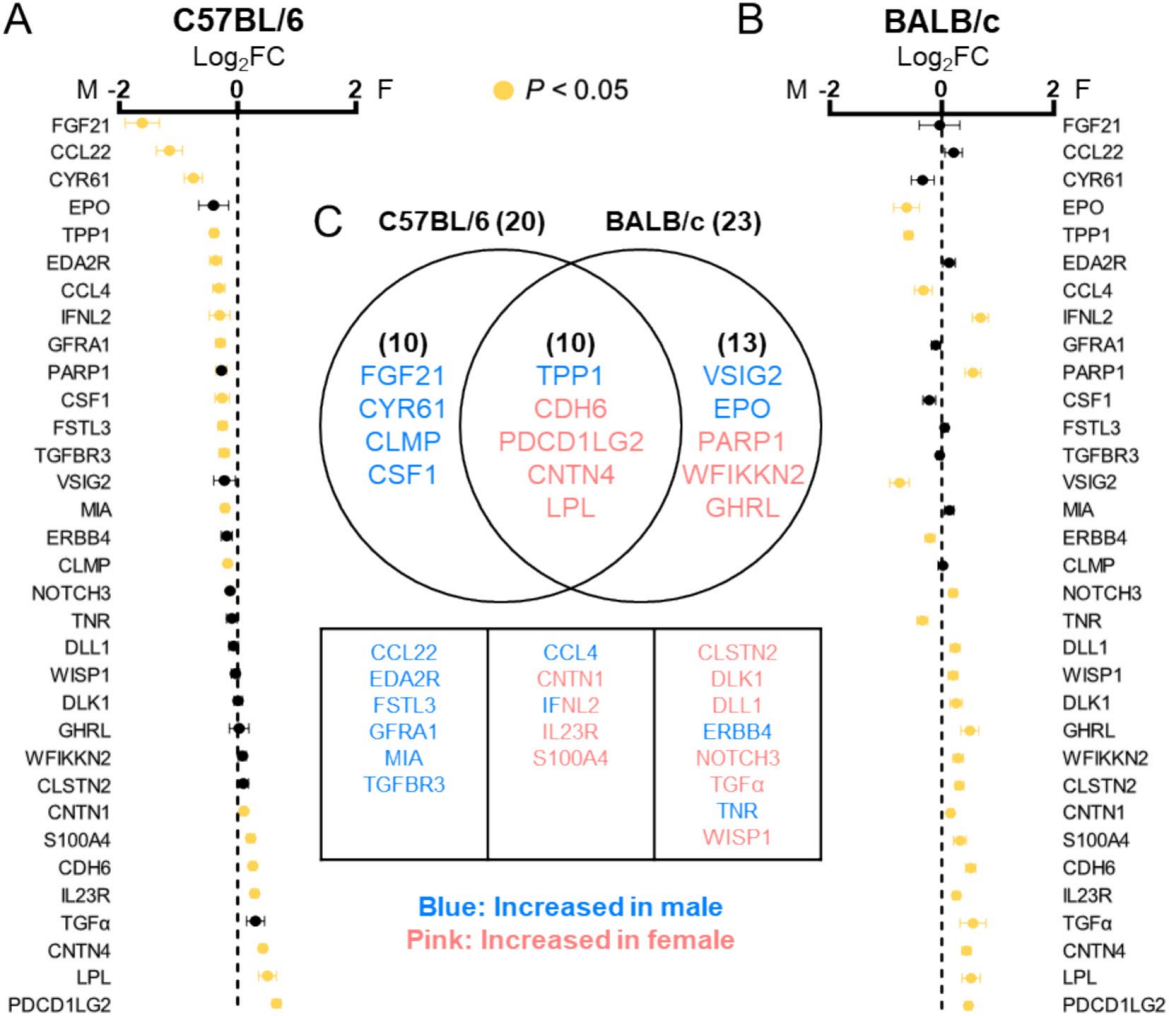
Sex-differential proteins in plasma. **(A, B)** Fold changes (FC) were calculated based on NPX values obtained from the Olink assay. **(A)** Comparison between C57BL/6 male and C57BL/6 female. **(B)** Comparison between BALB/c male and BALB/c female. Proteins with statistically significant differences (*P* < *0.05*) are highlighted in yellow. **(C)** Venn diagram and list illustrating the number and overlap of proteins showing sex differences in C57BL/6 and BALB/c mice.

**Fig. 5:**
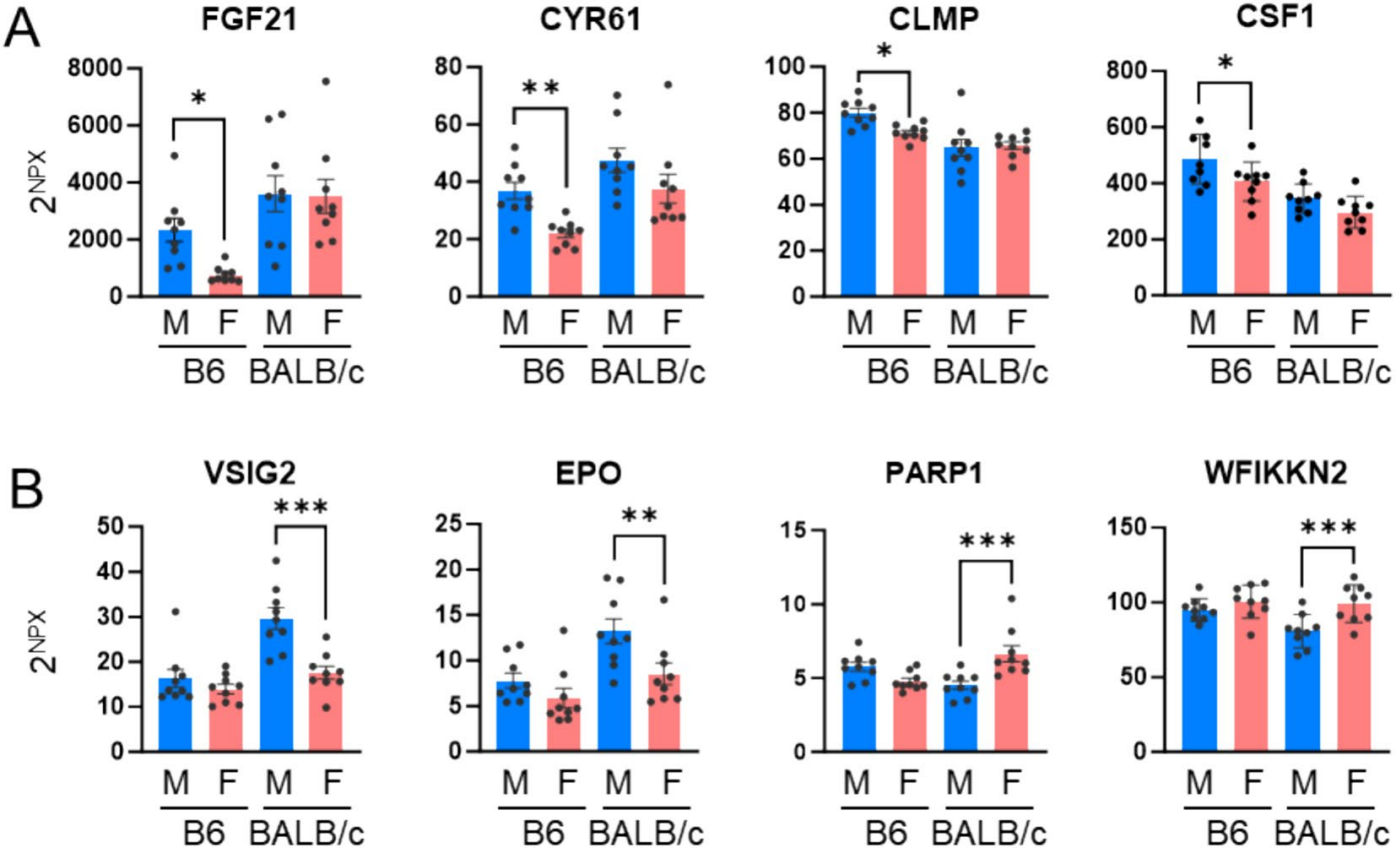
Representative plasma proteins that exhibit sex-differential expression differences. **(A)** Proteins showing sex differences only in C57BL/6 (B6) mice. **(B)** Proteins showing sex differences only in BALB/c mice. The data are denoted as the mean ± SEM. *P < 0.05, **P < 0.01, ***P < *0.001.*

Among the 87 analyzed proteins, 33 (37.9%) exhibited sex-differential expression. In C57BL/6 mice, 20 proteins showed sex-biased expression **(**Fig. 4A**)**, while 23 proteins were differentially expressed by sex in BALB/c mice **(**Fig. 4B**)**. Ten sex-differential proteins were commonly observed in both mouse strains **(**Fig. 4C**)**. Proteins showing sex-differential expression only in C57BL/6 included FGF21, CYR61, CLMP, and CSF1 **(**Fig. 5A**)**, while VSIG2, EPO, PARP1 and WFIKKN2 showed differential expression in BALB/c mice **(**Fig. 5B**)**.

### Proteins exhibiting conserved sex differences in both mice and humans

**Fig. 6:**
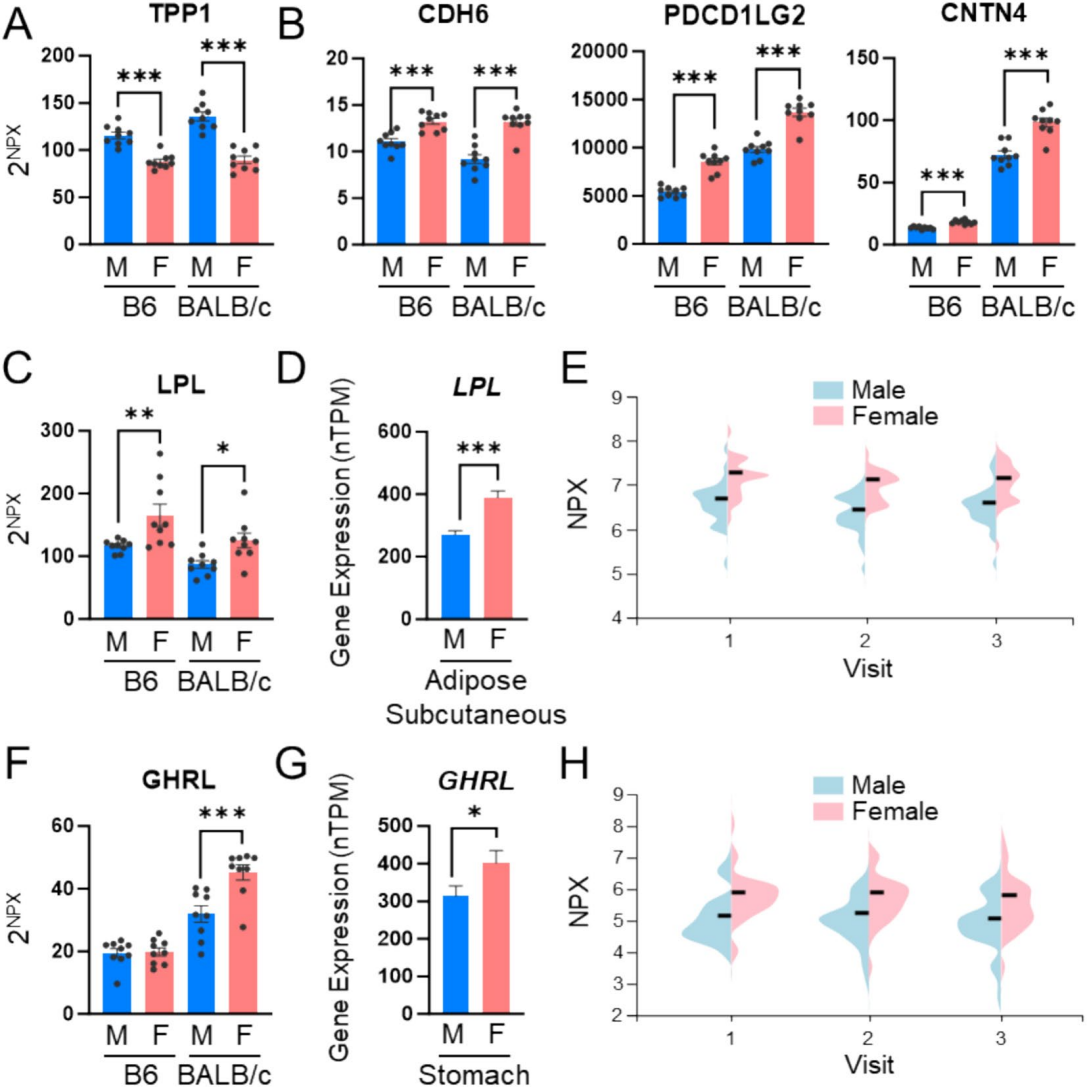
Sex-differential plasma protein expression conserved between mice and humans. **(A)** Representative proteins upregulated in males in the two mouse strains and humans. **(B)** Proteins upregulated in females in the two mouse strains and humans. **(C)** Expression levels of LPL in mouse plasma samples. **(D)** Expression levels of LPL in the human GTEx dataset. **(E)** Expression levels of LPL in human plasma samples. **(F)** Expression levels of GHRL in mouse plasma samples. **(G)** Expression levels of GHRL in the human GTEx dataset. **(H)** Expression levels of GHRL in human plasma samples. 76 participants (40 males and 36 females) visited the clinic at three time points: visit 1 (0 months), visit 2 (15–18 months), and visit 3 (21–24 months).

Previous studies using Olink technology have reported sex-differential protein expression in human plasma^28^, and we compared our mouse data with the human dataset. TPP1 showed higher plasma levels in males than females in both species **(**Fig. 6A**)**. Proteins with higher expression in females than in males in both species included CDH6, PDCD1LG2, and CNTN4 **(**Fig. 6B**)**. Lipoprotein lipase (LPL), which plays a crucial role in regulating plasma triglyceride levels and facilitating fatty acid deposition in adipose tissue^29^, was consistently more abundant in female plasma compared to males in both mouse strains **(**Fig. 6C**)**. This sex-differential pattern of LPL expression was also confirmed in human RNA-Seq data of GTEx dataset. LPL mRNA levels were significantly higher in the subcutaneous adipose tissue, the predominantly expressed tissue of LPL, of women **(**Fig. 6D**)**, and its plasma protein levels were also elevated in women **(**Fig. 6E**)** ^28^. Additional studies have similarly reported higher LPL levels in the serum of women^30^.

GHRL, also known as ghrelin, is a polypeptide mainly secreted by the stomach that stimulates appetite^31^. GHRL levels were elevated in female mice only in the BALB/c strain **(**Fig. 6F**)**. This female-enriched expression pattern of GHRL in mice was consistent with human RNA-Seq data of GTEx dataset. In human stomach tissue, GHRL expression was significantly higher in women compared to men **(**Fig. 6G**)**, and its plasma protein levels were also elevated in women **(**Fig. 6H**)**. Since both LPL and GHRL are involved in energy metabolism, these findings suggest the potential for sex-based differences in the pathophysiology of metabolic diseases in humans.

## Discussion

Plasma is a highly accessible and commonly used sample in both clinical and research settings, making it an advantageous source for biomedical study. To accurately understand sex-based differences in disease and improve patient outcomes, it is essential that researchers from various disciplines—such as medicine, nursing, and public health—engage more actively in sex-difference research^32^. In this study, we utilized Olink technology to sensitively detect sex- and strain differences in plasma protein expression in mice, suggesting that biological sex differences are widespread at the plasma proteome. We demonstrated that sex-dependent protein expression can vary depending on strain or, conversely, be conserved regardless of strain background.

Of particular interest, we identified sex differences in proteins involved in energy metabolism, such as LPL and GHRL. In a study involving 13,000 U.S. adults, although the average BMI between men and women was similar (27.9 and 28.2, respectively), the average body fat percentage was significantly higher in women (40.0%) compared to men (28.1%)^33^. These findings highlight substantial sex differences in lipid metabolism, and our data may provide molecular insights supporting this observation.

Our findings are consistent with previous human studies. In a study of healthy Japanese individuals aged 19 to 70 years (152 men and 225 women), plasma LPL levels were higher in women^34^. Similarly, in another study of Japanese participants (59 men and 148 women) who fasted for 12 h and showed no differences in BMI, women exhibited significantly higher LPL levels^35^. GHRL also demonstrated sex-specific differences. In 79 opposite-sex twin pairs (158 individuals), plasma ghrelin concentrations were significantly higher in women^36^. Circulating levels of LPL and ghrelin may be influenced by feeding status.

Since 55 proteins showed strain differences and 33 proteins exhibited sex differences, conventional animal experiments using only one sex or strain may overlook critical biological variations. Our findings suggest that including both male and female animals, and accounting for strain differences, can improve the reliability and reproducibility of experimental outcomes. Furthermore, researchers should consider selecting mouse strains that best recapitulate human sex-related biological differences for a given gene or protein of interest. Incorporating both sex and strain as key variables may increase the reproducibility of experiments and reduce clinical trial failure by enabling more representative preclinical models.

However, this study has some limitations. Only young, healthy 8-week-old mice were used, and thus age- or disease-associated changes in protein expression were not captured. Although we identified differences between mouse strains, the Human Protein Atlas dataset lacks racial and genetic variation information, precluding further comparison. Additionally, the two Olink panels used in this study contained a limited set of target proteins, and it is possible that other plasma proteins not included in the panels also exhibit sex- differential expression patterns.

For example, adipocytes from female C57BL/6 mice were reported to be more insulin-sensitive than those from males, with higher expression of multiple genes involved in glucose and lipid metabolism^37^. In addition, the activity of brown adipose tissue is greater in female than in male mice, which has been attributed to sex-specific differences in PGC-1α–mediated phospholipid synthesis^38^. Meanwhile, PM20D1, a regulator of thermogenesis, was observed to be expressed at higher levels in BALB/c mice than in C57BL/6 mice, owing to a polymorphism in the *Pm20d1* promoter^39^. These findings suggest that metabolic differences may exist between sexes and strains.

While our study focused on the plasma proteome, a multi-omics approach would be complementary. In a clinical lipidomic study of 1,086 fasted plasma samples from 364 individuals, sphingomyelins and ether-linked phospholipids were found to be more abundant in women^40^. Similarly, a metabolomic profiling study of 2,344 fasting plasma samples from 1,212 participants revealed that the temporal trajectories of 68 metabolites differed by sex^41^. With respect to gene expression in tissue level, 91.4% of FDA-approved drug target genes exhibit sex differences in at least one tissue^42^.

## Conclusions

This study identified sex- and strain differences in the expression of plasma proteins. These findings highlight the importance of considering both sex and strain in the discovery and application of protein-based biomarkers and also suggest the possibility of sex-based differences in disease progression and drug responses. Notably, the increased expression patterns of LPL and GHRL proteins in females were consistently observed in mouse plasma, as well as in human plasma and predominantly expressed tissue (subcutaneous adipose tissue and stomach, respectively). Such observations imply potential sex differences in energy and lipid metabolism and may serve as evidence for future studies aimed at elucidating the underlying sex-differential molecular mechanisms. In conclusion, considering sex as a critical biological variable is essential for biomarker discovery and paves the way toward precision medicine that enables personalized diagnosis and treatment. As more data on sex differences in various plasma proteins accumulate, they will serve as valuable resources to support and advance future research^43^.

## Data Availability

The datasets generated or analyzed in this study are available from the corresponding authors upon reasonable request.

## Abbreviations

CCL22: C–C motif chemokine 22
CDH6: Cadherin-6
CLMP: CXADR-like membrane protein
CNTN4: Contactin-4
CSF1: Macrophage colony-stimulating factor 1
CYR61: CCN family member 1
DLK1: Protein delta homolog 1
EDA2R: Tumor necrosis factor receptor superfamily member 2
EPCAM: Epithelial cell adhesion molecule
EPO: Erythropoietin
FGF21: Fibroblast growth factor 21
FST: Follistatin
GHRL: Appetite-regulating hormone
IFNA2: Interferon alpha-2
IL17A: Interleukin-17A
LPL: Lipoprotein lipase
PARP1: Poly [ADP-ribose] polymerase 1
PDCD1LG2: Programmed cell death 1 ligand 2
TPP1: Tripeptidyl-peptidase 1
VSIG2: V-set and immunoglobulin domain-containing protein 2
WFIKKN2: WAP, Kazal, immunoglobulin, Kunitz and NTR domain-containing protein 2

## References

[1] Deng, Y.-T. et al. Atlas of the plasma proteome in health and disease in 53,026 adults. *Cell* **188**(1), 253–71.e7 (2025). 39579765 10.1016/j.cell.2024.10.045

[2] Malmström, E. et al. Human proteome distribution atlas for tissue-specific plasma proteome dynamics. *Cell* **188**(10), 2810–22.e16 (2025). 40203824 10.1016/j.cell.2025.03.013

[3] Carrasco-Zanini, J. et al. Proteomic signatures improve risk prediction for common and rare diseases. *Nat. Med.* **30**(9), 2489–2498 (2024). 39039249 10.1038/s41591-024-03142-z PMC11405273

[4] Conforti, F. et al. Cancer immunotherapy efficacy and patients’ sex: a systematic review and meta-analysis. *Lancet Oncol.* **19**(6), 737–746 (2018). 29778737 10.1016/S1470-2045(18)30261-4

[5] Dong, M. et al. Sex differences in cancer incidence and survival: a pan-cancer analysis. *Cancer Epidemiol. Biomark. Prev.* **29**(7), 1389–1397 (2020). 10.1158/1055-9965.EPI-20-0036 32349967

[6] Mauvais-Jarvis, F. et al. Sex and gender: modifiers of health, disease, and medicine. *The Lancet.* **396**(10250), 565–582 (2020). 10.1016/S0140-6736(20)31561-0 PMC7440877 32828189

[7] Richardson, S. S. Sexes, species, and genomes: why males and females are not like humans and chimpanzees. *Biol. Philos.* **25**(5), 823–841 (2010).

[8] Kronzer, V. L., Bridges, S. L. Jr. & Davis, J. M. 3rd. Why women have more autoimmune diseases than men: An evolutionary perspective. *Evol Appl.* **14**(3), 629–633 (2021). 33767739 10.1111/eva.13167 PMC7980266

[9] Yan, Y. et al. X-linked ubiquitin-specific peptidase 11 increases tauopathy vulnerability in women. *Cell* **185**(21), 3913–30.e19 (2022). 36198316 10.1016/j.cell.2022.09.002 PMC9588697

[10] Kim, J. H. et al. Sex-dependent different clinicopathological characterization of Epstein-Barr virus-associated gastric carcinoma: a large-scale study. *Gastric Cancer* **27**(2), 221–234 (2024). 38212543 10.1007/s10120-023-01460-8 PMC10896815

[11] Kim, H. I., Lim, H. & Moon, A. Sex Differences in Cancer: Epidemiology. *Genetics and Therapy. Biomol Ther (Seoul).* **26**(4), 335–342 (2018). 29949843 10.4062/biomolther.2018.103 PMC6029678

[12] Kim, S. Y. et al. Sex-Biased Molecular Signature for Overall Survival of Liver Cancer Patients. *Biomol Ther (Seoul).* **28**(6), 491–502 (2020). 33077700 10.4062/biomolther.2020.157 PMC7585639

[13] Shin, J. Y., Jung, H. J. & Moon, A. Molecular Markers in Sex Differences in Cancer. *Toxicol Res.* **35**(4), 331–341 (2019). 31636844 10.5487/TR.2019.35.4.331 PMC6791665

[14] Kim, H., Park, J., Ahn, S. & Lee, H. The impact of sex/gender-specific funding and editorial policies on biomedical research outcomes: a cross-national analysis (2000–2021). *Sci. Rep.* **14**(1), 26599 (2024). 39496696 10.1038/s41598-024-77018-0 PMC11535369

[15] Beery, A. K. & Zucker, I. Sex bias in neuroscience and biomedical research. *Neurosci. Biobehav. Rev.* **35**(3), 565–572 (2011). 20620164 10.1016/j.neubiorev.2010.07.002 PMC3008499

[16] Becker, J. B., Prendergast, B. J. & Liang, J. W. Female rats are not more variable than male rats: a meta-analysis of neuroscience studies. *Biol. Sex Differ.* **7**(1), 34 (2016). 27468347 10.1186/s13293-016-0087-5 PMC4962440

[17] Prendergast, B. J., Onishi, K. G. & Zucker, I. Female mice liberated for inclusion in neuroscience and biomedical research. *Neurosci. Biobehav. Rev.* **40**, 1–5 (2014). 24456941 10.1016/j.neubiorev.2014.01.001

[18] Kaluve, A. M., Le, J. T. & Graham, B. M. Female rodents are not more variable than male rodents: A meta-analysis of preclinical studies of fear and anxiety. *Neurosci. Biobehav. Rev.* **143**, 104962 (2022). 36402227 10.1016/j.neubiorev.2022.104962

[19] Loeb, J. M., Hendee, W. R., Smith, S. J. & Schwarz, M. R. Human vs Animal Rights. *In Defense of Animal Research. JAMA.* **262**(19), 2716–2720 (1989). 2810604

[20] King, A. J. The use of animal models in diabetes research. *Br. J. Pharmacol.* **166**(3), 877–894 (2012). 22352879 10.1111/j.1476-5381.2012.01911.x PMC3417415

[21] Lee, H. et al. It is time to integrate sex as a variable in preclinical and clinical studies. *Exp Mol Med.* **50**(7), 1–2 (2018). 30038313 10.1038/s12276-018-0122-1 PMC6056479

[22] Lilue, J. et al. Sixteen diverse laboratory mouse reference genomes define strain-specific haplotypes and novel functional loci. *Nat. Genet.* **50**(11), 1574–1583 (2018). 30275530 10.1038/s41588-018-0223-8 PMC6205630

[23] Takeda, K., Haczku, A., Lee, J., Irvin, C. & Gelfand, E. Strain dependence of airway hyperresponsiveness reflects differences in eosinophil localization in the lung. *American Journal of Physiology-Lung Cellular and Molecular Physiology.* **281**(2), L394–L402 (2001). 11435214 10.1152/ajplung.2001.281.2.L394

[24] Ezzatvar, Y., Ramírez-Vélez, R., Izquierdo, M. & García-Hermoso, A. Racial differences in all-cause mortality and future complications among people with diabetes: a systematic review and meta-analysis of data from more than 2.4 million individuals. *Diabetologia* **64**(11), 2389–2401 (2021). 34455457 10.1007/s00125-021-05554-9

[25] Humayun, M., Mukasa, L., Ye, W., Bates, J. H. & Yang, Z. Racial and Ethnic Disparities in Tuberculosis Incidence, Arkansas, USA, 2010–2021. *Emerg Infect Dis.* **30**(1), 116–124 (2024). 38146997 10.3201/eid3001.230778 PMC10756389

[26] Temple, R. & Stockbridge, N. L. BiDil for Heart Failure in Black Patients: The U.S. Food and Drug Administration Perspective. *Ann. Intern. Med.* **146**(1), 57–62 (2007). 17200223 10.7326/0003-4819-146-1-200701020-00010

[27] Tannenbaum, C., Ellis, R. P., Eyssel, F., Zou, J. & Schiebinger, L. Sex and gender analysis improves science and engineering. *Nature* **575**(7781), 137–146 (2019). 31695204 10.1038/s41586-019-1657-6

[28] Zhong, W. et al. Next generation plasma proteome profiling to monitor health and disease. *Nat. Commun.* **12**(1), 2493 (2021). 33941778 10.1038/s41467-021-22767-z PMC8093230

[29] Voshol, P. J., Rensen, P. C. N., van Dijk, K. W., Romijn, J. A. & Havekes, L. M. Effect of plasma triglyceride metabolism on lipid storage in adipose tissue: Studies using genetically engineered mouse models. *Biochimica et Biophysica Acta (BBA) - Molecular and Cell Biology of Lipids.* **1791**(6), 479–485 (2009). 19168150 10.1016/j.bbalip.2008.12.015

[30] Hirano, T., Nishioka, F. & Murakami, T. Measurement of the serum lipoprotein lipase concentration is useful for studying triglyceride metabolism: comparison with postheparin plasma. *Metabolism* **53**(4), 526–531 (2004). 15045703 10.1016/j.metabol.2003.10.021

[31] Kojima, M. et al. Ghrelin is a growth-hormone-releasing acylated peptide from stomach. *Nature* **402**(6762), 656–660 (1999). 10604470 10.1038/45230

[32] Kim, N. Application of sex/gender-specific medicine in healthcare. *Korean J Women Health Nurs.* **29**(1), 5–11 (2023). 37037446 10.4069/kjwhn.2023.03.13 PMC10085661

[33] Li, C., Ford, E. S., Zhao, G., Balluz, L. S. & Giles, W. H. Estimates of body composition with dual-energy X-ray absorptiometry in adults. *Am. J. Clin. Nutr.* **90**(6), 1457–1465 (2009). 19812179 10.3945/ajcn.2009.28141

[34] Watanabe, H. et al. Preheparin serum lipoprotein lipase mass level: the effects of age, gender, and types of hyperlipidemias. *Atherosclerosis* **145**(1), 45–50 (1999). 10428294 10.1016/s0021-9150(99)00012-x

[35] Wu, B. et al. Different Associations of Trunk and Lower-Body Fat Mass Distribution with Cardiometabolic Risk Factors between Healthy Middle-Aged Men and Women. *International journal of endocrinology.* **2018**(1), 1289485 (2018). 29531527 10.1155/2018/1289485 PMC5817354

[36] Makovey, J., Naganathan, V., Seibel, M. & Sambrook, P. Gender differences in plasma ghrelin and its relations to body composition and bone–an opposite-sex twin study. *Clin. Endocrinol.* **66**(4), 530–537 (2007). 10.1111/j.1365-2265.2007.02768.x 17371471

[37] Macotela, Y., Boucher, J., Tran, T. T. & Kahn, C. R. Sex and depot differences in adipocyte insulin sensitivity and glucose metabolism. *Diabetes* **58**(4), 803–812 (2009). 19136652 10.2337/db08-1054 PMC2661589

[38] Takeuchi, A. et al. Sex difference in BAT thermogenesis depends on PGC-1α–mediated phospholipid synthesis in mice. *Nat. Commun.* **16**(1), 6072 (2025). 40659621 10.1038/s41467-025-61219-w PMC12259864

[39] Simoes, M. R. et al. Bidirectional shifts in Pm20d1 expression impact thermogenesis and metabolism. *Mol. Med.* **31**, 283 (2025). 40877813 10.1186/s10020-025-01345-9 PMC12395774

[40] Medina, J. et al. Clinical lipidomics reveals high individuality and sex specificity of circulatory lipid signatures: a prospective healthy population study. *J. Lipid Res.* **66**(5), 100780 (2025). 40112951 10.1016/j.jlr.2025.100780 PMC12022646

[41] Darst, B. F., Koscik, R. L., Hogan, K. J., Johnson, S. C. & Engelman, C. D. Longitudinal plasma metabolomics of aging and sex. *Aging (Albany NY).* **11**(4), 1262 (2019). 30799310 10.18632/aging.101837 PMC6402508

[42] Suh, Y., Lee, J. G. & Kim, K. E. Analysis of sex-differential gene expression on the target of approved drug. *Sci. Rep.* **15**(1), 26989 (2025). 40707586 10.1038/s41598-025-12342-7 PMC12289949

[43] Lee, S. K. Sex as an important biological variable in biomedical research. *BMB Rep.* **51**(4), 167–173 (2018). 29429452 10.5483/BMBRep.2018.51.4.034 PMC5933211

